# Recent Evolutions of Multiple Sequence Alignment Algorithms

**DOI:** 10.1371/journal.pcbi.0030123

**Published:** 2007-08-31

**Authors:** Cédric Notredame

**Affiliations:** Whitehead Institute, United States of America

An ever-increasing number of biological modeling methods depend on the assembly of an accurate multiple sequence alignment (MSA). These include phylogenetic trees, profiles, and structure prediction. Assembling a suitable MSA is not, however, a trivial task, and none of the existing methods have yet managed to deliver biologically perfect MSAs. Many of the algorithms published these last years have been extensively described [1–3], and this review focuses only on the latest developments, including meta-methods and template-based alignment techniques.

The purpose of an MSA algorithm is to assemble alignments reflecting the biological relationship between several sequences. Computing exact MSAs is computationally almost impossible, and in practice approximate algorithms (heuristics) are used to align sequences, by maximizing their similarity. The biological relevance of these MSAs is usually assessed by systematic comparison with established collections of structure-based MSAs (“gold standards”; for review see [4]). Since only a few sequences have known structures, the accuracy measured on the references is merely an estimation of how well a package may fare on standard datasets. Gold standards have had a considerable effect on the evolution of MSA algorithms, refocusing the entire methodological development toward the production of structurally correct alignments. Their use has also coincided with a notable algorithmic harmonization, most MSA packages being now based on the “progressive algorithm” [5]. This greedy heuristic assembly algorithm involves estimating a guide tree (rooted binary tree) from unaligned sequences, and then incorporating the sequences into the MSA with a pairwise alignment algorithm while following the tree topology. The progressive algorithm is often embedded in an iterative loop where the guide tree and the MSA are reestimated until convergence. Most MSA packages reviewed here [6–18] follow this canvas, albeit more or less extensively adapted for improved performances [1–3].

The scoring schemes used by the pairwise alignment algorithm are arguably the most influential component of the progressive algorithm. They can be divided in two categories: matrix- and consistency-based. Matrix-based algorithms such as ClustalW [14], MUSCLE [6], and Kalign [19] use a substitution matrix to assess the cost of matching two symbols or two profiled columns. Although profile statistics can be more or less sophisticated, the score for matching two positions depends only on the considered columns or their immediate surroundings. By contrast, consistency-based schemes incorporate a larger share of information into the evaluation. This result is achieved by using a recipe initially developed for T-Coffee [10] and inspired by Dialign overlapping weights [20]. Its principle is to compile a collection of pairwise global and local alignments (primary library) and to use this collection as a position-specific substitution matrix during a regular progressive alignment. The aim is to deliver a final MSA as consistent as possible with the alignments contained in the library. Many recent packages have built upon this initial framework. For instance, PCMA [15] decreases T-Coffee computational requirements by prealigning closely related sequences. ProbCons [7] uses Bayesian consistency and fills the primary library using the posterior decoding of a pair hidden Markov model. The substitution costs are estimated from this library using Bayesian statistics. MUMMALS [17] combines the ProbCons scoring scheme with the PCMA strategy, while including secondary structure predictions in its pair hidden Markov model. The most accurate flavors of MAFFT [8] (i.e., the GNS and LNS modes) use a T-Coffee–like evaluation. A majority of studies indicate that consistency-based methods are more accurate than their matrix-based counterparts [4], although they typically require an amount of CPU time *N* times higher than simpler methods (*N* being the number of sequences). Most of these methods are available online, either as downloadable packages or as online Web services (Table 1).

**Table 1:**
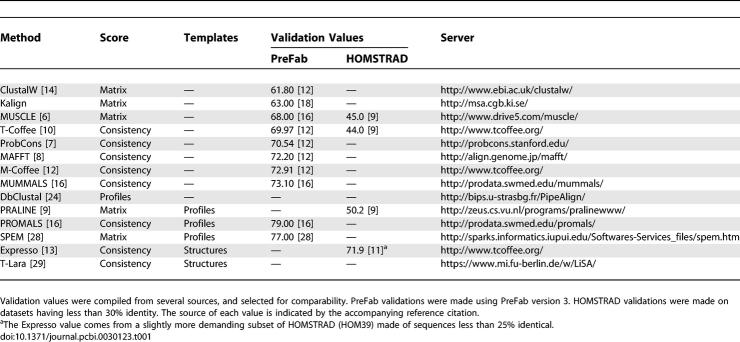
Summary of the Methods Described in the Review

The wealth of available methods and their increasingly similar accuracies makes it harder than ever to objectively choose one over the others. Consensus methods such as M-Coffee [12] provide an interesting framework to address this problem. M-Coffee is a consensus meta-method based on T-Coffee. Given a sequence dataset, it fills up the library by using various MSA methods to compute alternative alignments. T-Coffee then uses this library to compute a final MSA consistent with the original alignments. When combining eight of the most accurate and distinct MSA packages, M-Coffee produces 67% of the time a better MSA than ProbCons, the best individual method [12]. Aside from its ease of extension, M-Coffee's main advantage is its ability to estimate the local consistency between the final alignment and the combined MSAs (CORE index [21]; Figure 1). This useful index has been shown to be well-correlated with the MSAs' structural correctness [21,22]. M-Coffee is not, however, the ultimate answer to the MSA problem, and its limited performances on remote homologs suggest that further improvement using only sequence information remains an elusive goal. Progress is nonetheless needed, and, at this point, the most promising approach is probably to incorporate within the datasets any information likely to improve the alignments, such as structural and homology data. Template-based alignment methods [13] follow this approach.

**Figure 1:**
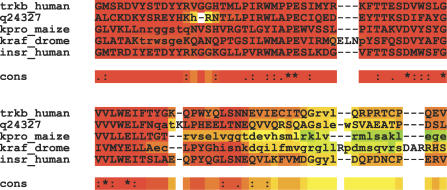
Typical Output of M-Coffee This output was obtained on the kinase1_ref5 BaliBase dataset, by combining MUSCLE, MAFFT, POA, Dialign-T, T-Coffee, ClustalW, PCMA, and ProbCons with M-Coffee. Correctly aligned residues (as judged from the reference) are uppercase; noncorrect ones are lowercase. The color of each residue indicates the agreement of the individual MSAs with respect to the alignment of that specific residue. Red indicates residues aligned in a similar fashion among all the individual MSAs; blue indicates very low agreement between MSAs. Dark yellow, orange, and red residues can be considered to be reliably aligned.

Structural extension was initially described by Taylor [23]. The principle is fairly straightforward (Figure 2) and involves identifying with BLAST a structural template in the Protein Data Bank for each sequence, aligning the templates using a structure superposition method, and mapping the original sequences onto their template's alignment. The resulting sequence alignments are compiled in the primary library and used by a consistency-based method to compute the final MSA. Homology extension was originally introduced in the DbClustal package [24] and works along the same lines, using a profile rather than a structure. PSI-BLAST is used to build a profile for each sequence, and these profiles are used as templates to generate better sequence alignments, thanks to the evolutionary information they contain. The only difference between homology and structure extension is the templates' nature and the associated alignment method. This generic approach can easily be extended to any kind of template. For instance, Expresso [13] uses SAP [25,26] and FUGUE [27] to align structural templates identified by a BLAST against the Protein Data Bank. PROMALS [17], PRALINE [9], and SPEM [28] make a profile–profile alignment with PSI-BLAST profiles used as templates. In PRALINE and PROMALS, the profile can be complemented with a secondary structure prediction in an attempt to improve the alignment accuracy. PROMALS uses ProbCons Bayesian consistency to fill its library with the posterior decoding of a pair hidden Markov model. T-Lara [29] uses RNA secondary structure predictions as templates and fills a T-Coffee library with the Lara pairwise algorithm. With the exception of PRALINE and SPEM, which use a regular progressive algorithm, most template-based methods described here are consistency-based (some of them taking advantage of T-Coffee modular structure). Their main advantage is increased accuracy. Recent benchmarks on PROMALS (Table 1) show that homology extension results in a ten-point improvement over existing methods. Likewise, structure-based methods such as Expresso produce alignments much closer to the structural references than do any of their sequence-based counterparts. One must, however, be careful not to over-interpret validation values like that given for Expresso in Table 1, since both the reference and the Expresso alignments were computed using the same structural information.

**Figure 2:**
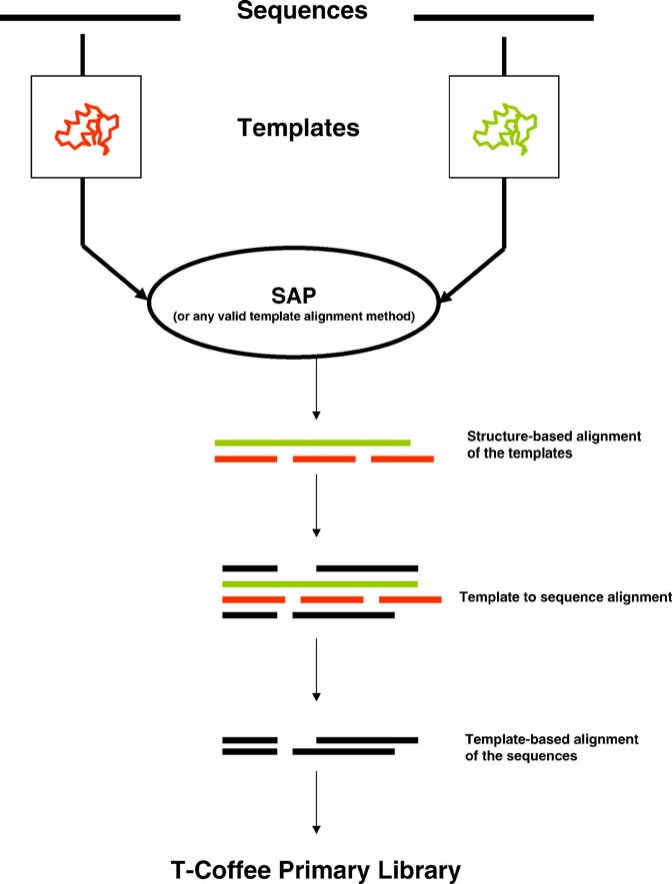
Framework of a Template-Based Method Structural templates are first identified, mapped onto the sequences, and aligned using SAP. The sequence–template mapping is then used to guide the alignment of the original sequences. This alignment is integrated into the library that is used to compute the final MSA.

This last point raises the important issue of method validation and benchmarking. A recent study [4] shows that with the exception of artificial datasets, benchmarks carried out on most reference databases tend to deliver compatible results. It also suggests that the best methods have become indistinguishable, except when considering remote homologs (less than 25% identity). Unfortunately, remote homologs are poorly suited to generating reference alignment, owing to the fact that their superposition often yields alternative sequence alignments that are structurally equivalent [30]. However, one can bypass the reference alignment stage by directly comparing the evaluated alignment to some idealized 3-D superposition. Such an alignment-independent evaluation has been described and used by several authors [17,31,32]. Another trend, not well accounted for by current reference collections, is the alignment of very large datasets. While many new methods incorporate special algorithms for aligning several hundred sequences [6,8,18], current reference databases do not allow the evaluation of very large datasets, thus making it unclear how the published accuracies scale with the number of sequences. While this last issue could probably be satisfyingly solved in the current benchmarking framework, another problem remains that is much harder to address. All the existing validation approaches have in common their reliance on the “one size fits all” assumption that structurally correct alignments are the best possible MSAs for modeling any kind of biological signal (evolution, homology, or function). A report on profile construction [33] has recently challenged this view by showing that structurally correct alignments do not necessarily result in better profiles. Likewise, it may be reasonable to ask whether better alignments always result in better phylogenetic trees, and, more systematically, to question and quantify the relationship between the accuracy of MSAs and the biological relevance of any model drawn upon them.

In this review, I have presented some of the latest additions to the MSA computation arsenal. An interesting milestone has been the development of meta-methods able to seamlessly combine the output of several methods. Aside from easing the user's work, the main advantage of these consensus methods is probably the local estimation of reliability they provide (Figure 1). Using this estimation to filter out unreliable regions has already proven useful in homology modeling [34] and could probably be used further. The main improvement reported here, however, is probably the notion of template-based alignment. Template-based alignment is more than a trivial extension of consistency-based methods. Under this new model, the purpose of an MSA is not to squeeze a dataset and extract all the information it may contain, but rather to use the dataset as a starting point for exploring and retrieving all the related information contained in public databases. This information is to be used not only for mapping purposes, but also for driving the MSA computation. Such a usage of sequence information makes template-based methods a real paradigm shift and a major step toward global biological data integration. 

## Abbreviations

MSA: multiple sequence alignment
